# Early transition from short-term romosozumab to antiresorptive therapies: analysis of 26 cases

**DOI:** 10.1007/s11657-025-01598-1

**Published:** 2025-07-31

**Authors:** Judith Everts-Graber, Serge Ferrari, Albrecht Popp, Magaly Hars, Mathias Wenger, Sven Oser, Ueli Studer, Christian Steiner, Hans-Rudolf Ziswiler, Gernot Schmid, Stephan Reichenbach, Thomas Lehmann, Olivier Lamy, Elena Gonzalez-Rodriguez

**Affiliations:** 1 Department of Rheumatology and Immunology, Inselspital, Bern University Hospital, University of Bern, Anna-Seiler-Haus, Freiburgstrasse 20, 3010 Bern, Switzerland; 2 OsteoRheuma Bern, Bahnhofplatz 1, Bern, Switzerland; 3 Department of Diabetes, Endocrinology, Nutritional Medicine and Metabolism, Inselspital, Bern University Hospital, University of Bern, Bern, Switzerland; 4 Division of Bone Diseases Geneva University Hospital and Faculty of Medicine Geneva, Geneva, Switzerland; 5 Zentrum Für Rheuma- Und Knochenerkrankungen, Klinik Im Park, Hirslanden Zurich, Switzerland; 6 Department of Rheumatology, Lucerne Regional Hospital, Lucerne, Switzerland; 7 Institute for Social and Preventive Medicine, University of Bern, Bern, Switzerland; 8 Centre Interdisciplinaire Des Maladies Osseuses, Lausanne University Hospital and University of Lausanne, Lausanne, Switzerland

**Keywords:** Osteoporosis, Romosozumab, Bone mineral density, Bone formation

## Abstract

***Summary*:**

This multicentre, retrospective case series analysed bone mineral density (BMD) changes in 26 patients who switched early from romosozumab (3–10 months) to antiresorptives. BMD gains over 12 months were similar to those in patients (*n* = 99) completing the full 12-month course.

**Background:**

Romosozumab is typically administered for a duration of 12 months before transitioning to antiresorptive therapies. This study analysed the bone mineral density (BMD) changes of patients who were prematurely switched to an antiresorptive regimen.

**Methods:**

This multicentre, retrospective case series investigated the BMD response to romosozumab administered for 3 to 10 months, followed by subsequent antiresorptive therapy, across four bone centres in Switzerland. BMD measurements at the lumbar spine, total hip and femoral neck were conducted at the initiation of romosozumab and again 12 months later. The study compared the BMD changes in patients who received short-term romosozumab with those in a cohort of patients who completed the full 12-month course.

**Results:**

Twenty-six patients (25 postmenopausal women and one man, median age 73 years [interquartile range: 65, 81]) were enrolled from February 2022 to December 2024. They received a median of six romosozumab injections (range: 3 to 10) and were prematurely switched to antiresorptives (14 to denosumab, 11 to zoledronate and one to alendronate) due to possible side effects or adverse events. Over 12 months, BMD increased by 13.5% [8.6, 16.6] at the lumbar spine, 2.9% [0.3, 7.3] at the total hip and 3.2% [0.4, 7.8] at the femoral neck, without significant differences compared with the cohort of 99 patients who received 12 months of romosozumab therapy. In both the short- and full-duration romosozumab treatment groups, significantly lower BMD responses were observed in patients who were pretreated with antiresorptives compared with those who were treatment naïve.

**Conclusion:**

In patients who underwent an early switch from romosozumab to antiresorptive therapy, BMD responses during the first year were similar to those in patients who completed the full 12-month romosozumab treatment. However, the subsequent changes in BMD, when all patients are receiving antiresorptive therapy, remain to be determined.

## Introduction

Romosozumab is a monoclonal antibody that inhibits sclerostin, a protein that is produced by osteocytes and suppresses bone formation. By blocking sclerostin, romosozumab activates the Wnt signaling pathway, leading to a dual effect on bone: increasing bone formation and decreasing bone resorption [1, 2]. This leads to substantial gains in bone mineral density (BMD) at both the lumbar spine and hip [3–5], and reduces the incidence of fractures in postmenopausal women [4, 5]. In Switzerland, romosozumab is approved for treating postmenopausal women (and exceptionally men) at high risk of fragility fractures. Reimbursement is provided for patients who have experienced a major osteoporotic fracture in the last 24 months and have a T-score of −3.5 SD or lower, those with two major osteoporotic fractures, and those without fractures but with a very high fracture risk. While romosozumab is typically administered for 12 months, some patients are switched to antiresorptive therapy prematurely due to possible side effects or cardiovascular events. In clinical trials, the anabolic effect of romosozumab, as indicated by serum levels of N-terminal propeptide of type 1 procollagen (P1NP), peaks within a few weeks and then gradually returns to baseline [6]. Serum levels of C-terminal telopeptide of type I collagen (CTX), which is a marker of bone resorption, decline rapidly after treatment begins, then show a slight increase, and finally decrease further over 12 months, but they remain higher overall than under treatment with alendronate [6]. The short anabolic effects of romosozumab are explained by the increased expression of Wnt inhibitors in bone, such as Dkk-1 and Sost itself [7, 8], and raise the question of whether a shorter course of romosozumab, followed by a potent antiresorptive, could achieve similar effects to continuing romosozumab for the full 12 months. This case series aims to describe BMD changes in patients who received a short course of romosozumab (fewer than 10 monthly injections) before switching to an antiresorptive regimen.

## Methods

### Setting and outcome

This retrospective case series was conducted at four locations in Switzerland, including three university hospitals and one large outpatient centre. All four sites record data on patients receiving romosozumab in different databases, one of them being the Swiss Osteoporosis Registry, which recently published the first results regarding the effectiveness of romosozumab therapy in patients who were pretreated with antiresorptives or were treatment naïve [9]. The primary objective of this multicentre case series was to assess changes in BMD (at the lumbar spine, total hip and femoral neck) within 12 months in patients who received fewer than 10 monthly romosozumab injections and who were prematurely switched to antiresorptives for various reasons. These BMD changes were compared with those in the previously described cohort that received romosozumab for 12 months [9]. This cohort was derived, at least in part, from the same institutions and was treated by the same physicians following similar standard-of-care practices. Further, this study analysed the correlation between romosozumab treatment duration and BMD changes at both the lumbar spine and total hip among patients who received fewer than 10 injections.

### Study population

The patients reviewed in this study were treated with romosozumab (210 mg monthly) for 3 to 10 months, followed by antiresorptive therapy, and evaluated by DXA between February 1, 2022, and December 31, 2024. Postmenopausal women and men aged ≥ 50 years who received ≤ 10 monthly romosozumab injections and who underwent DXA and vertebral fracture assessment (VFA) on the days of the first romosozumab injection and around 12 months later were eligible for the study. Thus, the observed BMD changes reflect not only the effects of the short course of romosozumab therapy but also, to some extent, those of the subsequent antiresorptive treatment. Both DXA measurements in each patient were performed using the same device.

The study protocol was reviewed by three local ethics committees that waived the need for a full ethics authorisation (swissethics, req 2024–00480). All patients provided written informed consent for further use of their health-related data.

### Statistical analysis

The primary outcome was the difference between the BMD changes over 12 months in patients who received 12 months of romosozumab therapy and in those who were prematurely switched to an antiresorptive treatment. Continuous variables are summarised as median with interquartile range and were compared using the Wilcoxon-Mann–Whitney test, while categorical variables are shown as number with percentage and were compared using Fisher’s exact test. To investigate the association between the duration of romosozumab therapy (short-term versus 12-month duration) and the change in BMD after romosozumab treatment, we performed linear regression that first included only romosozumab treatment as the covariate, and then also the baseline T-score at the lumbar spine and femoral neck. The correlation between the romosozumab duration (in months) and the BMD change at the lumbar spine or total hip was evaluated with Spearman’s test. All analyses were conducted using Stata 16.0 (StataCorp LLT, College Station, Texas), and figures were created with Graph Pad Prism Version 10.0 (GraphPad Software Inc., La Jolla, California).

## Results

### Patient description

Between February 1, 2022 (the date of the first enrolment of a patient receiving Evenity® (romosozumab), which was approved in Switzerland in August, 2020), and December 31, 2024, a total of 26 patients at the four study sites in Switzerland received 3 to 10 romosozumab injections and were prematurely switched to antiresorptive therapies. Patients were recruited consecutively, and no individuals with shorter romosozumab durations were intentionally excluded from this case series. Patients received calcium and vitamin D supplementation as needed.

The characteristics of all 26 patients are described in Table 1, including the duration of romosozumab therapy, the subsequent antiresorptive drug and the reason for romosozumab discontinuation. Of note, the majority of patients (*n* = 17, 65%) had received treatment before initiating romosozumab, most commonly with bisphosphonates (*n* = 15). Additionally, one patient was switched from raloxifene to romosozumab, and another from teriparatide (after 2 months) to romosozumab.

**Table 1:** Individual data of patients with short-term romosozumab therapy (*n* = 26)

Case #	Age	Sex	# Romo	BMI	Prior Th	# Prior Fx	LS T-score	TH T-score	FN T-score	%Δ LS	%Δ TH	%Δ FN	LS 2	TH 2	FN 2	Subseq. AR	Reason for Romo Discontinuation
1	60	f	4	18.3	1	4	−2.5	−2.8	−2.5	6.8%	3.1%	1.0%				Dmab	Arthralgias
2	62	f	9	18.4	1	0	−3.3	−1.1	−1.9	13.5%	3.8%	4.6%	−2.4	−0.8	−1.6	Dmab	Local skin reactions
3	66	f	8	22.3	0	1	−4.1	−1.5	−1.8	22.4%	10.3%	9.1%	−2.7	−0.8	−1.3	Dmab	Multiple diffuse symptoms
4	87	f	5	24.6	1	2		−2.3	−2.4	3.9%	4.1%	5.4%	1.8	−1	−2.2	Dmab	Stroke
5	73	f	3	17.6	1	3	−5.0	−3.3	−3.4	15.8%	7.6%	11.2%	−4.3	−2.9	−2.9	Dmab	Angina pectoris
6	68	f	6	25.5	0	3	−5.4	−2.2	−2.3	13.8%	9.4%	2.1%	−4.8	−1.7	−2.2	Dmab	Angina pectoris
7	65	f	6	23.4	1	2	−3.0	−1.6	−1.6	8.9%	1.0%	2.3%	−2.4	−1.6	−1.5	Dmab	Thoracic pain
8	83	f	8	19	1	3	−5.4	−4.2	−3.8	20.5%	1.5%	2.7%	−4.5	−4.2	−3.7	Dmab	Hypercalcemia
9	61	f	4	22.2	0	1	−3.8	−1.5	−1.8	16.2%	6.4%	8.0%	−2.9	−1.1	−1.3	ZOL	Facial paresthesias
10	88	f	6	22.2	0	5	−3.6	−3.3	−3.5		2.3%	0.0%		−3.4	−3.6	ZOL	Fatigue
11	81	f	2	26.8	1	2	−3.9	−1.5	−2.1	16.0%	−4.3%	−3.1%	−1.8	−2.3	−2.9	ZOL	Arthralgias and headache
12	71	f	5	20.5	1	5	−5.9	−3.5	−3	47.5%*	11.7%	8.8%	−4	−2.9	−2.6	ZOL	Multiple diffuse symptoms
13	57	f	3	17.3	0	1	−2.9		−4.4				−3	−4	−4.2	ALN	Local and systemic symptoms
14	80	f	8	22.8	1	2	−3.5	−3	−3.4	21.5%	3.0%	2.9%	−2.1	−2.9	−3	Dmab	Erythema after injections
15	65	m	6	17	0	3	−3.6	−3.1	−3.1	44.5%*	8.9%	−0.8%	−0.7	−2.7	−3.1	ZOL	Oral cancer
16	71	f	8	22.2	1	3	−4.8	−2.8	−3.7	6.6%	0.0%	7.0%	−4.5	−2.8	−3.4	ZOL	Eczema
17	81	f	6	23.1	0	6	−0.9	−1.2	−1.4	17.4%	5.0%	4.9%	0.6	−0.8	−1.1	ZOL	Asymptomatic ischemic cerebral lesions
18	80	f	9	19.5	1	5	−4.1	−2.5	−3.1	10.7%	−2.2%	−9.7%	−3.5	−2.6	−3.5	Dmab	Skin rash
19	81	f	3	24	1	1	−3	−2.8	−3.1	11.2%	2.6%	−2.6%	−2.3	−2.6	−3.2	ZOL	Anterior myocardial infarction
20	65	f	9	21.6	1	2	−3.5	−4.2	−3.5	15.8%	0.1%	2.2%	−2.5	−4.2	−3.5	ZOL	Constipation
21	87	f	5	23.7	1	5	−0.7	−2	−2.7	5.3%	1.8%	3.6%	−0.3	−1.9	−2.5	Dmab	Confirmation of old ischemic stroke
22	79	f	8	22.7	1	2	−4.1	−2.8	−3.3	11.5%	−0.2%	3.4%	−3.4	−2.8	−3.2	Dmab	Hypertensive crisis
23	79	f	10	21.5	1	4		−2.3	−2.6		−1.8%	0.2%		−2.4	−2.6	ZOL	Cutaneous adverse drug reactions
24	72	f	6	23.8	1	3	−0.8			8.20%			−0.1			Dmab	Myalgias
25	61	f	7	22.7	0	5	−4.2	−3	−3.1	21.1%	29.5%	18.4%	−3	−1.5	−1.9	Dmab	Recurrent back pain
26	81	f	6	20.9	0	2	−2.5	−3.1	−2.9	9.7%	2.8%	8.1%	−1.7	−2.8	−2.8	ZOL	Myalgias

Prior Therapy (Th): 1 = yes, 0 = no. LS = lumbar spine, TH = total hip, FN = femoral neck. LS 2, TH 2 and FN 1: Follow-up T-scores 12 months after romosozumab initiation. Subseq. *AR* Subsequent antiresorptive therapy,* ZOL* Zoledronate, *Dmab* Denosumab, *ALN* Alendronate

*Values excluded from analysis

Age, sex, body mass index (kg/m^2^), prevalent vertebral and non-vertebral fractures, prior antiresorptive treatment and baseline T-scores at different locations are shown in Table 2, comparing the 26 patients with short-term romosozumab treatment with the 99 patients who received the full 12-month course. No significant differences were observed in terms of age, sex or proportion of pretreated patients. While the baseline T-scores at the lumbar spine and total hip were similar in the two groups, patients with short-term romosozumab therapy had a lower baseline BMD at the femoral neck.

**Table 2:** Patient characteristics at baseline

	12 months (*n* = 99)	Short term (*n* = 26)	*p*-value
Age (years)	71 [65, 76]	73 [65, 81]	0.2
Sex			
Female	92 (93%)	25 (96%)	
Male	7 (7%)	1 (4%)	0.99
BMI (kg/m2)	22 [20, 25]	22 [19 to 24]	0.81
Prior antiresorptive treatment	77 (78%)	17 (65%)	0.21
Number of prior fractures	2.0 [1.0, 4.0]	3.0 [2.0, 4.0]	0.13
T-score lumbar spine	−3.2 ([−3.7 to −2.1]	−3.6 [−4.2 to −2.9]	0.15
T-score total hip	−2.4 [−2.8 to −1.9]	−2.8 [−3.1 to −1.7]	0.21
T-score femoral neck	−2.4 [−2.9 to −1.9]	−3.0 [−3.4 to −2.2]	**0.02**

Characteristics of patients receiving short-term (3 to 10 month) versus 12-month romosozumab treatment

*BMI* body mass index

### BMD Changes under short-term versus 12-month romosozumab therapy

**Fig. 1:**
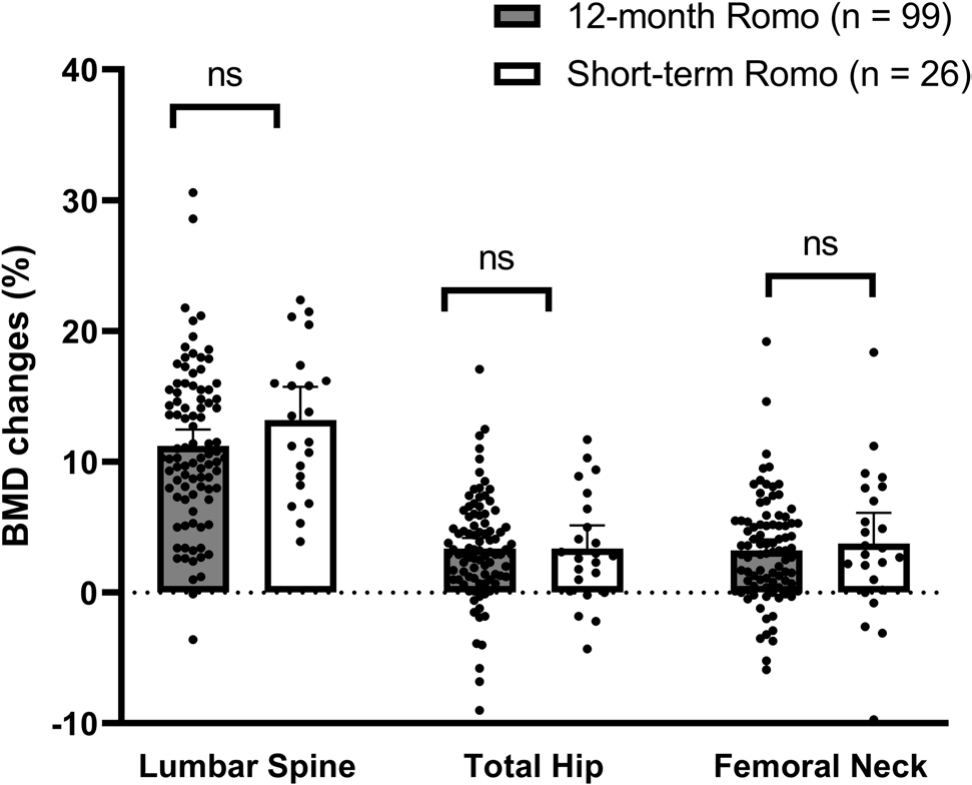
BMD changes under 12-month romosozumab therapy (grey) versus short-term romosozumab and subsequent antiresorptive therapy (white) at the lumbar spine, total hip and femoral neck. Data are presented as mean ± 95% CI

The BMD changes at the lumbar spine, total hip and femoral neck within 12 months (median 12.0, interquartile range: 10.8, 12.0) after initiation of romosozumab therapy are shown in Fig. 1, comparing the 26 patients who received short-term romosozumab treatment with the 99 patients who received the full 12-month course. In patients with a short romosozumab treatment duration (and subsequent antiresorptive therapy), BMD increased by 13.5% [8.6, 16.6] at the lumbar spine, 2.9% [0.3, 7.3] at the total hip and 3.2% [0.4, 7.8] at the femoral neck. There were no significant differences compared with the cohort of 99 patients who received 12 months of romosozumab therapy, who demonstrated BMD gains of 10.3% [7.5, 15.5] at the lumbar spine, 3.1% [1.1, 5.8] at the total hip and 3.1% [0.5, 5.3] at the femoral neck (lumbar spine p = 0.14, total hip p = 0.99, femoral neck p = 0.63). Of note, two patients with short-term romosozumab therapy who had very high BMD gain at the lumbar spine (> 44%) were excluded to prevent outliers from skewing the results. However, a sensitivity analysis including these patients did not significantly alter the overall findings. Furthermore, the comparison of BMD gains following short- versus standard-duration romosozumab therapy, adjusted for baseline T-scores at the lumbar spine and femoral neck, yielded similar results (adjusted p-values: lumbar spine 0.72, total hip 0.21, femoral neck 0.26).

### Subanalysis of BMD changes in pretreated versus treatment-naïve patients

**Fig. 2:**
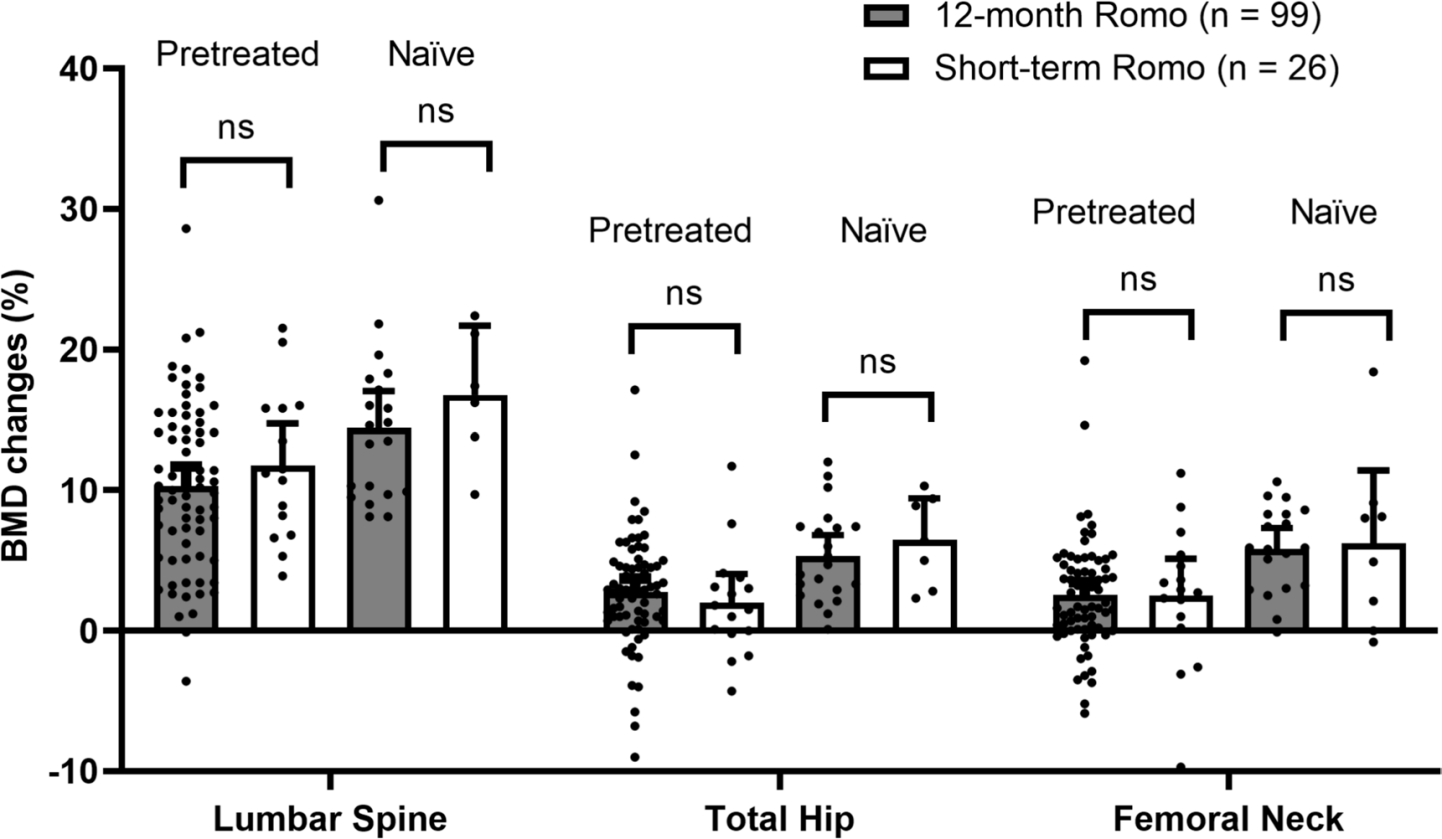
BMD changes under short-term (*n* = 26) and 12-month (*n* = 99) romosozumab therapy depending on prior antiresorptive treatment (*n* = 77 and *n* = 17, respectively) or lack thereof (*n* = 22 and *n* = 9, respectively). Data are presented as mean ± 95% CI

The majority of the 26 patients (*n* = 17, 65%) with short-term romosozumab therapy were pretreated with an antiresorptive, mainly a bisphosphonate (*n* = 15). This was also the case among the 99 patients who received 12 months of romosozumab therapy (*n* = 77, 78%, 75 with a prior bisphosphonate). Figure 2 shows the BMD changes in patients with short-term romosozumab therapy (*n* = 26) and those with 12-month treatment (*n* = 99), depending on prior antiresorptive treatment status. Notably, within the short-term romosozumab group, treatment-naïve patients experienced significantly greater BMD gains compared with pretreated patients, both at the lumbar spine (+ 5.5% [4.7, 5.2], p = 0.05) and the total hip (+ 5.9% [3.7, 8.7], p = 0.005). However, no statistically significant differences in BMD changes were observed between patients who received short- versus long-term romosozumab therapy, even when stratified by prior treatment status.

### Correlation between romosozumab treatment duration and BMD gains

**Fig. 3:**
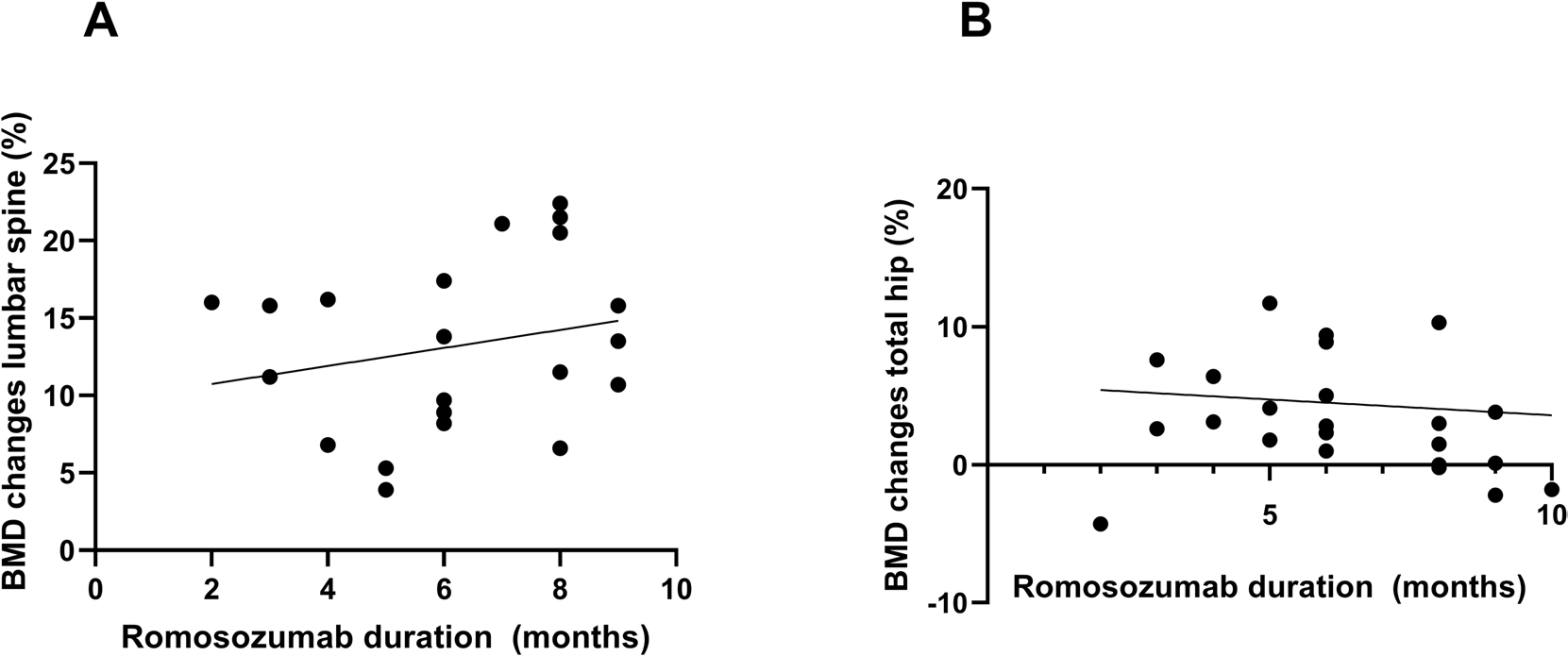
Correlation between romosozumab treatment duration (months) and BMD changes at the lumbar spine (**A**) and total hip (**B**)

Among patients with fewer than 10 injections, there was no significant correlation between the romosozumab treatment duration (in months) and either the BMD change at the lumbar spine (r^2^ 0.005, p = 0.73) or that at the total hip (r^2^ 0.005, p = 0.33) (Fig. 3).

## Discussion

This retrospective case series examined BMD changes in patients who received romosozumab therapy but were prematurely switched to antiresorptives after a median of 6 months (range: 3 to 10 months) due to side effects or adverse events. No significant differences were observed when comparing their BMD changes to those in a cohort of 99 patients who completed the full 12-month romosozumab course. In both groups, however, patients pretreated with antiresorptives exhibited lower BMD gains at the lumbar spine and hip than those who were treatment naïve, an effect previously reported in clinical trials [10] and real-world studies [9, 11, 12]. The gains observed in both groups were comparable to those reported in a meta-analysis of placebo-controlled trials after 6 and 12 months of romosozumab treatment: 9.3% and 12.7% at the lumbar spine, 2.7% and 4.4% at the total hip and 2.3% and 4.0% at the femoral neck, respectively [13]. While that meta-analysis identified higher BMD gains at 12 months than at 6 months, no statistical comparison between these treatment durations has been performed. Notably, between 58% (at the femoral neck) and 73% (at the lumbar spine) of the total absolute BMD gain occurred within the first 6 months, suggesting that shortening the treatment duration does not lead to a proportional loss of BMD gain.

This early BMD gain can be explained by the effect of romosozumab on modelling-based bone formation. Histomorphometrical analyses of iliac crest bone biopsies showed early modelling-based bone formation on both trabecular and cortical bone surfaces within 2 months, resulting from a reactivation of bone lining cells and recruitment from a progenitor pool [14, 15]. After 12 months, indices of bone resorption (such as erosin depth) remained significantly lower than those following placebo treatment [16]. This demonstrates that 12 months of romosozumab induces a positive bone balance at remodeling units early in treatment, subsequently leading to overall gains. This could potentially also be achieved by an early switch to potent antiresorptives before 12 months. However, it remains unclear if this would provide the same improvements in bone strength and anti-fracture efficacy as the full 12-month treatment duration.

Another uncertainty concerns the trajectory of BMD changes and fracture risk reduction after all patients have transitioned to antiresorptive therapy. Data from the FRAME study suggested that the benefits of romosozumab extended beyond the first year of treatment [17]. In the second year, when all patients were receiving denosumab, overall fracture rates remained low. However, during this second year, fracture rates were consistently lower in patients who had received romosozumab in year 1 compared with those who had initially received placebo [17]. It remains to be seen whether long-term BMD improvements might be blunted in romosozumab patients who switch to antiresorptives early, particularly those with prior antiresorptive treatment. However, our findings are reassuring, suggesting that patients who discontinue romosozumab due to side effects and promptly transition to antiresorptive therapy can still achieve meaningful BMD gains.

This observation also suggests new treatment perspectives, particularly for patients with bone conditions in which excessive osteoblastic stimulation is a concern, such as individuals with cancer and osteoblastic bone metastases. In this case series, some patients discontinued romosozumab following cardiovascular events, in light of the ongoing debate regarding its potential cardiovascular risk [18]. Therefore, the proportion of cardiovascular events observed in our study should not be interpreted as indicative of the incidence or risk of such events attributable to romosozumab, nor should it be generalised beyond this specific context. Nevertheless, if romosozumab is discontinued for any reason, subsequent antiresorptive therapy is warranted to preserve BMD gains and prevent bone loss.

### Limitations

Our findings are limited by the observational study design, small sample size and unequal group distribution. Additionally, BMD gains tended to be greater in patients with lower baseline BMD, which may partially explain the lumbar spine BMD increases of over 40% observed in two patients in the short-term romosozumab group. To minimize bias, we excluded these two outliers in a sensitivity analysis; however, the results remained unchanged. Lastly, the duration of short-term romosozumab therapy ranged from 3 to 10 months, meaning that treatment responses may have differed between patients who were treated for just 3 months versus 8–9 months, even though the overall BMD changes were comparable in both groups and no significant correlation was found between BMD changes and romosozumab treatment duration. Notably, the lack of correlation between romosozumab treatment duration and BMD increase may be attributed to the small sample size and should be interpreted with caution.

## Conclusion

In patients who made an early switch from romosozumab to antiresorptive therapy, BMD responses during the first 12 months were similar to those in patients who completed the full 12-month romosozumab regimen. However, the BMD changes in subsequent years, once all patients are receiving antiresorptive therapy, have yet to be determined. Therefore, no definitive conclusions can be drawn at this time about whether a shorter romosozumab regimen is as effective as the standard 12-month course. In addition, the observational design limits the strength of our conclusions; therefore, our findings should be considered hypothesis-generating and interpreted with caution.

## Data Availability

Data can be made available to qualified researchers upon reasonable request.
